# The Role of Fatty Acid Metabolites in Vaginal Health and Disease: Application to Candidiasis

**DOI:** 10.3389/fmicb.2021.705779

**Published:** 2021-07-02

**Authors:** Silke Baldewijns, Mart Sillen, Ilse Palmans, Paul Vandecruys, Patrick Van Dijck, Liesbeth Demuyser

**Affiliations:** ^1^Laboratory of Molecular Cell Biology, Institute of Botany and Microbiology, KU Leuven, Leuven-Heverlee, Belgium; ^2^VIB-KU Leuven Center for Microbiology, Leuven, Belgium

**Keywords:** vaginal candidiasis, fatty acid metabolites, metabolome, microbiome, *Candida albicans*

## Abstract

Although the vast majority of women encounters at least one vaginal infection during their life, the amount of microbiome-related research performed in this area lags behind compared to alternative niches such as the intestinal tract. As a result, effective means of diagnosis and treatment, especially of recurrent infections, are limited. The role of the metabolome in vaginal health is largely elusive. It has been shown that lactate produced by the numerous lactobacilli present promotes health by limiting the chance of infection. Short chain fatty acids (SCFA) have been mainly linked to dysbiosis, although the causality of this relationship is still under debate. In this review, we aim to bring together information on the role of the vaginal metabolome and microbiome in infections caused by *Candida*. Vulvovaginal candidiasis affects near to 70% of all women at least once in their life with a significant proportion of women suffering from the recurrent variant. We assess the role of fatty acid metabolites, mainly SCFA and lactate, in onset of infection and virulence of the fungal pathogen. In addition, we pinpoint where lack of research limits our understanding of the molecular processes involved and restricts the possibility of developing novel treatment strategies.

## The Vaginal Metabolome

### The Composition of the Vaginal Metabolome

The vagina is a muscular structure lined with epithelial cells, connecting the uterus with the outside world (Farage and Maibach, 2006; O’Hanlon et al., 2013). A major component of the vaginal niche are its secretions, also known as the vaginal fluid. This vaginal fluid is not only composed of contributions from the host herself as vaginal transudate, secretions from glands residing in the vaginal area, such as Bartholin’s and Skenes’s glands, epithelial cells, residual urine, mucus from the cervix and endometrial fluids, also metabolites by the vaginal microbiota contribute significantly to its composition (Paavonen, 1983; Morrison and Preston, 2016). The vaginal microbiota consists of over 50 different microbial species coexisting in a balanced environment, establishing intricate connections with the host and each other (Oakley et al., 2008; Ma et al., 2012). This microbial composition of the vagina fluctuates intra- and inter-individually, especially between women from different geographical environments and age (Farage and Maibach, 2006). Most of these microbial species exists in a mutualistic relationship with the host. However, some are opportunistic pathogens with the potential to cause infections and even life-threatening diseases (Sobel, 1990). To establish a balanced connection with its environment and host, microorganisms produce metabolites. These metabolites do not only play an important role as a chemical barrier to protect the host against pathogens but also function in the maintenance of the overall homeostasis of the vaginal niche (Aldunate et al., 2015). The predominant metabolites in this niche are the short chain fatty acids (SCFA) (Cunha et al., 2017; Tachedjian et al., 2017), amines, organic acids, amino acids, nitrogenous bases, and monosaccharides (Vitali et al., 2015).

Women produce approximately 6 g of vaginal fluid per day, with 0.5–0.75 g present at any given time (Owen and Katz, 1999). The vaginal fluid is mainly composed of fatty acids, proteins, salts and carbohydrates (Table 1). However, in this regard it should be noted that large inter- and intra-individual variation of these components exists. Several studies determined the concentrations of ions, such as sodium (1.38 g/L), potassium (0.987 g/L), calcium (0.120 g/L), and chloride (2.13 g/L) in the vagina (Levin and Wagner, 1977; Wagner and Levin, 1978, 1980; Owen and Katz, 1999), as well as the presence of proteins in the vaginal fluid and found them to be present in a range from 0.015 to 0.026 g/L (Bdallah and de Vargas-Linares, 1971; Raffi et al., 1977; Huggins and Preti, 1981). In healthy women, median glucose levels in vaginal fluid were reported to be 5, 2 mM (0.94 g/L). However, inter-individual variation can be observed (between 0.2 and 149 mM) (Ehrstrom et al., 2006). Concentrations of many other carbohydrates have not yet been quantified. The concentrations of fatty acids present in the vaginal fluid were determined by different research teams using varying techniques, resulting in consensus concentrations of 2 and 1 g/L for lactate and acetic acid, respectively (Oberst and Plass, 1936; Preti and Huggins, 1975; Huggins and Preti, 1976; Preti et al., 1979). The relative ratio of L- and D-lactate present in the vagina strongly depends on the microbial composition. While epithelial cells can only produce the L-isomer, certain bacteria can produce both (Boskey et al., 2001). On average, the proportion of D-lactic acid is reported to be around 55% of the total lactic acid present. Glycerol and urea appear to be present in the vaginal fluid at concentrations of 0.16 and 0.4 g/L, respectively. Glycogen concentrations found in the vaginal fluid differ significantly between different research reports, ranging between 0.1 and 32 g/L, possibly depending on the specific characteristics of the tested individuals (Lapan and Friedman, 1950; Stamey and Timothy, 1975; Mirmonsef et al., 2016). These glycogen levels were inversely correlated with pH and progesterone levels.

**TABLE 1:** Composition of vaginal fluid.

**Vaginal metabolites**		**Concentration (g/L)**
*Ions*	Na^+^	1.38
	K^+^	0.987
	Ca^2+^	0.120
	Cl^–^	2.13
*Proteins*		0.015–0.026
*Carbohydrates*		Unknown
*Fatty acids*	Lactic acid	2
	Acetic acid	1
*Glycerol*		0.16
*Urea*		0.4
*Glycogen/glucose*		4.4–15*

* Depends on the specific characteristics of the tested individuals.

*Details and references are given in the main text.*

This review highlights the role of fatty acid metabolites in vaginal health and disease with an application on vulvovaginal candidiasis. We focus specifically on SCFA, which are fatty acids with less than six carbon atoms that include acetic acid, propionic acid and butyric acid. Because of their specific relevance in vaginal infections, we also address particularly relevant medium chain fatty acids (MCFA), with 6–11 carbon atoms, and the intermediates of fatty acid metabolism, such as lactic acid and succinic acid (Brody, 1998).

### Factors That Influence the Vaginal Metabolome

Many factors appear to influence the composition of the vaginal metabolome either direct or by altering the composition of the vaginal microbiome. Changes in the vaginal microbiome consequently lead to changes in the metabolic profile. Ceccarani et al. (2019) investigated the metabolic profiles of samples derived from healthy patients (HP) and patients suffering from *Chlamydia trachomatis* (CT), vulvovaginal candidiasis (VVC), and bacterial vaginosis (BV). They found a sharp decrease in lactate concentration in CT, VVC, and BV conjointly with an increased vaginal pH, which is a marker of dysbiosis. Additionally, they concluded that proliferation of diverse bacterial genera that play a role in establishment of the vaginal dysbiosis was associated with the increased presence of SCFA such as butyrate, propionate and acetate. Additionally, upon treatment of the vaginal dysbiosis, such as BV, with antibiotics, the metabolic profiles restore to levels similar to healthy vaginas (Laghi et al., 2014).

Ethnicity influences the composition of the vaginal microbiome as well, thereby shaping the metabolic profile. Studies performed by Ravel et al. (2011) showed differences in the composition of the vaginal microbiome between four self-named ethnicities (white, Asian, black, and Hispanic). The vaginal niche of Asian and white women is dominated by higher levels of *Lactobacillus* species compared to Hispanic and black women (Ravel et al., 2011). This coincided with lower pH values. However, contrary to the stigma in which the prevalence of high quantities of lactobacilli and low pH are said to define a “healthy” vagina, a vaginal microbiome that is not dominated by *Lactobacillus* appears frequently and can be defined as “normal” in black and Hispanic women (Zhou et al., 2007). The reasons for this difference in vaginal microbiome and consequently in vaginal metabolome, remains unknown. This variation along ethnic backgrounds could be a consequence of genetic predisposition or geographical and behavioral differences (Ravel et al., 2011). The latter includes the number of sexual partners, the use of contraception devices, showering, eating habits, clothing, and smoking (Schwebke, 2009). It was shown that women suffering from BV were significantly less likely to use condoms or hormonal contraception (Smart et al., 2004). A study by Nelson et al. (2018) proved that cigarette smoking is associated with changes in vaginal metabolome. They found that nicotine and the resulting degradation metabolites were significantly increased in the vaginal niche of smoking individuals (Nelson et al., 2018). In addition, the presence of breakdown products of several drugs such as painkillers, cocaine and antidepressants in the metabolic profiles was demonstrated in vaginal fluids (Nelson et al., 2018). In addition to the role of the vaginal microbiome in metabolome composition, also the activity of the host immune system affects the composition of the vaginal secretions (Ravel et al., 2011).

The age and hormone levels of women also have a significant impact on the composition of the vaginal microbiome and consequently on the metabolome. Until puberty, estrogen and thus also glycogen levels remain low, which causes the vagina to be dominated with anaerobic micro-organisms (Farage and Maibach, 2006). From puberty on, estrogen levels rise, leading to production of cervicovaginal secretions and colonization by high numbers of lactobacilli, with increasing concentrations of lactate as a result. Upon pregnancy, the vagina remains dominated by lactobacilli but is characterized by lower richness and diversity than in non-pregnant women (DiGiulio et al., 2015). Following menopause, estrogen and glycogen levels decrease, causing the *Lactobacillus* dominance to decrease and eventually cease (Cauci et al., 2002; Gupta et al., 2006). Additionally, throughout the menstrual cycle estrogen and glycogen levels vary, ranging from low levels during menses to the highest levels before ovulation (Sjöberg et al., 1988; Gajer et al., 2012). This variation might also explain the differences in microbiome composition (Nunn and Forney, 2016).

### Analysis of Vaginal Microbiome and Metabolome

In the human vagina, the microbiota plays an important role in preventing vaginal infections, like BV and VVC (Sobel and Chaim, 1996; Fredricks et al., 2005). The last decade, the bacterial and metabolite composition of the vaginal microbiota has been studied to improve diagnosis, to identify biomarkers of disease and to characterize the complex interplay between the microbiota and the host metabolism (Srinivasan S. et al., 2015; Vitali et al., 2015; Watson and Reid, 2018; Ceccarani et al., 2019; Oliver et al., 2020).

Traditionally, vaginal infections are diagnosed by subjecting vaginal fluids to microscopic evaluation, pH measurements as well as visual and olfactory evaluation (Biagi et al., 2009). BV is often diagnosed using the Nugent scoring system, possibly in combination with the Amsel criteria (Amsel et al., 1983; Nugent et al., 1991). Nugent scoring relies on gram staining to determine the bacterial composition of vaginal secretions (Nugent et al., 1991). The Amsel criteria refer to clinical signs and symptoms associated with the infection, such as the occurrence of the vaginal discharge, the vaginal pH, the presence of clue cells (these are squamous epithelial cells covered with adherent bacteria) and amine production. VVC can be diagnosed through the observation of clinical symptoms e.g., itching or vaginal whitish discharge, microscopy and colony appearance as well as pigmentation in chromogenic culture medium (Biagi et al., 2009; Baron et al., 2013; Eddouzi et al., 2013; Maheronnaghsh et al., 2016; Vecchione et al., 2017). Unfortunately, these methods lack precision and accuracy due to diverse morphology of vaginal microorganisms, subjectivity in microscopic examination and non-diagnosis of BV in women with asymptomatic infections (Schwebke et al., 1996; Chaijareenont et al., 2004; Beverly et al., 2005). Moreover, culture-based microbiome assessments are hampered by the diverse growth requirements or even uncultivable nature of various strains (2003—The uncultured microbial majority—Rappé). *Lactobacillus iners* exemplifies the bias that culture conditions exert, as this organism is only able to proliferate on blood agar, in contrast to other lactobacilli that are able to grow on Mann Rogosa Sharpe (MRS) agar (Vaneechoutte, 2017). As a result, its role in the vaginal microbial flora was unknown prior to 1999. Since then, multiple cultivation-independent studies have demonstrated the predominance of *L. iners* in the vagina of healthy women. To avoid these situations, more rapid and reliable diagnostic tools are needed (Oliver et al., 2020).

During the last decade, combinations of genomic, proteomic, and metabolomic techniques were developed to characterize micro-organisms and correlate their presence to specific metabolic profiles and virulence associated parameters (Bai et al., 2012; Yeoman et al., 2013; McMillan et al., 2015; Vitali et al., 2015; Oliver et al., 2020). A well-established method to study the composition of the microbiome is the analysis of the 16S ribosomal RNA gene amplicon sequences (Thies et al., 2007; Biagi et al., 2009; Zhou et al., 2009; Caporaso et al., 2011; Borgogna et al., 2020). This sequence is present in all bacteria and contains both regions of sequence conservation and sequence heterogeneity. Therefore, it can be amplified with broad range of PCR primers and enables the identification of bacteria or infer phylogenetic relationships (Hugenholtz and Pace, 1996; Pace, 1997; Baker et al., 2003; Schmidt, 2006; Weng et al., 2006). Besides Sanger sequencing, electrophoretic fingerprinting techniques like denaturing gradient gel electrophoresis (DGGE) and terminal restriction fragment length polymorphism (T-RFLP) can be used for analysis (Muyzer et al., 1993; Thies et al., 2007). T-RFLP is a rapid and reliable technique that allows more differentiation of the microbiota than DGGE (Horz et al., 2001). Fluorescence *in situ* hybridization (FISH), using nucleic acid probes, oligonucleotides complementary to rRNA gene targets, labeled with a fluorescent tag, can give additional information about the microbiota (Fredricks et al., 2005; Srinivasan and Fredricks, 2008). Moreover, by performing next generation sequencing of barcoded 16S rRNA amplicons, large amounts of data can be obtained to characterize the vaginal microbiome (Ravel et al., 2011; Bai et al., 2012; Brotman et al., 2014; Hang et al., 2014; Ceccarani et al., 2019; Borgogna et al., 2020). However, sample collection, storage, nucleic acid extraction as well as PCR amplification, amplicon sequencing and the selected bioinformatics analysis can affect the accuracy and resolution of these metagenomic approaches (Hamady and Knight, 2009; Kuczynski et al., 2011; Bai et al., 2012). For identification of *Candida* species, genomic regions of the rDNA genes can be sequenced. Comparison of these sequences using basic local alignment search tool (BLAST) algorithms^1^ or the MycoBank database^2^ can provide an accurate species identification. Amplification and subsequent sequencing of the D1/D2 domain of the 28S rDNA can serve the same goal (Chen et al., 2000; Leaw et al., 2006; Cornet et al., 2011; Rezaei-Matehkolaei et al., 2016). Unfortunately, sequencing of clinical isolates is time-consuming and not yet standardized (Lacroix et al., 2014). In the last years, matrix-assisted laser desorption/ionization time-of-flight mass spectrometry (MALDI-TOF MS) was used for microorganism identification, both at species and genus levels. This technique shows high precision and efficiency while allowing a low error rate and ensuring rapid analysis. To improve the identification rate, however, spectral databases require a regular update (Bader et al., 2011; Haigh et al., 2011; Bille et al., 2012; Bader, 2013; Sandrin et al., 2013; De Carolis et al., 2014; Lacroix et al., 2014; Foschi et al., 2017; Hendrickx, 2017; Oliver et al., 2020).

One of the techniques for metabolomic analysis is nuclear magnetic resonance (^1^H-NMR). H-NMR is a high-throughput, rapid, non-destructive method with low running costs that allows chemotype characterization of microorganisms. By analyzing the presence and the quantity of small molecule metabolites simultaneously, this tool can be used for researchers to determine the effects caused by perturbations on the host’s metabolic profiles. Nevertheless, the use of standardized conditions, growth media and pure cultures of isolates are required to ensure reproducible and accurate identification (Himmelreich et al., 2003, 2005, 2017; Sandrin et al., 2013; Vitali et al., 2015; Foschi et al., 2017; Oliver et al., 2020). Metabolomic profiles of vaginal secretions can also be determined by gas (GC-MS) and liquid chromatography and mass spectrometry (LC-MS). Metabolites are identified by comparing the corresponding spectra to a database with reference metabolite standards. Standards of metabolites of interest can also be used to confirm identities during a run. Although both methods can measure the concentration in the samples, it is not possible to differentiate whether the metabolite is derived from the host or the microorganisms (McMillan et al., 2015; Nelson et al., 2018; Borgogna et al., 2020). Gas chromatography is the most widely used method for SCFA analysis and is, given its sensitivity, well suited for accurate analysis of samples with low concentrations of SCFA, such as human samples (McGrath et al., 1992; Hoving et al., 2018). Since SCFA are volatile molecules, collection and proper storage of the samples is critical for reproducibility. They should be kept frozen and vacuum dried. Before processing the samples, pretreatment, distillation, ultrafiltration, and extraction are important for a rapid qualitative and quantitative SCFA determination in biological samples. To remove the protein fraction in the samples and simultaneously obtain the maximum yield of small molecules after purification, a series of organic and aqueous extractions can be conducted, followed by removal of the organic solvent (Zhao et al., 2006; Fiorini et al., 2015; Hoving et al., 2018; Nelson et al., 2018; Zhang C. et al., 2020). However, one of the most critical steps in the GC-MS analysis of SCFA is the derivatization (e.g., by silylation or alkylation) to improve separation. In the past, SCFA were converted into their methyl ester, or trimethylsilyl esters. Nowadays, other derivatization reagents are used, such as the alkylation reagents pentafluorobenzyl bromide (PFBBr), bistrimethyl-silyl-trifluoroacetamide (BSTFA), tert-butyl dimethylsilyl (TBDMS), propyl chloroformate or isobutyl chloroformate. More recently, the use of benzyl chloroformate (BCF) was suggested to obtain better and more reproducible results (Quehenberger et al., 2011; Tomcik et al., 2011; Zheng et al., 2013; Kloos et al., 2014; Gao and Xu, 2015; Furuhashi et al., 2018; Hoving et al., 2018; Nelson et al., 2018; Kim et al., 2019). GC-MS aided detection of lactic acid requires derivatization using alkyl chloroformates, like methyl chloroformate (MCF), ethyl chloroformate (ECF), propyl chloroformate (PCF), and isobutyl chloroformate (IBCF) (Sobolevsky et al., 2004; Qiu et al., 2007; Zampolli et al., 2007; Stefanelli et al., 2018; Zhang et al., 2018). For all molecules, GC provides separation based on affinity to the mobile gas phase vs. the stationary capillary phase. Molecules with different properties will elute at a different retention time. The mass spectrometer will allow identification of the separated fractions, based on differences in mass and charge. Both LC and GC-based methods can be modified to specifically determine the ratio of L- and D-isomers of lactic acid in a sample. Either using a chiral stationary phase or conversion of the enantiomers with a chiral product and consequent separation on a non-chiral column can be opted for (Inoue et al., 2006). Since the specific detection of both enantiomers requires additional instrumentation, many studies in which the metabolome was investigated, do not distinguish between both isomers.

## The Role of Fatty Acid Metabolites in the Healthy Vaginal Niche

### Origin of Vaginal Fatty Acid Metabolites

#### Microbial Fatty Acid Metabolites

In the last decades much attention has been given to the investigation of the human microbiome in government-backed projects, such as the MetaHIT project (METAgenomics of the Human Intestinal Tract) and the Human Microbiome Project (Hmp et al., 2009; Ehrlich and Consortium, 2011). Although the importance of the micro-, and to a lesser extent, mycobiome and its metabolites on gut health has been established, little attention has been given to the vaginal microbiota and its role in intimate health (Cui et al., 2013). This is likely due to the gender health care gap which encompasses the general underrepresentation and underfunding of research on female-specific conditions, such as VVC (Council, 2015). Although the vaginal microbiome of healthy women is dominated by bacteria (10^10^–10^11^ bacterial cells/ml), its exact composition is unique (Mitchell et al., 2015; Chen C. et al., 2017). Archaea, protists, fungi and viruses are often present, but in lower numbers compared to bacteria (Belay et al., 1990; Ravel et al., 2011; Drell et al., 2013; Kusdian and Gould, 2014; Bradford and Ravel, 2017; Zhang et al., 2021). The largest group of healthy women (±70–80%) show vaginal microbiomes dominated by the aerotolerant anaerobic *Lactobacillus* bacteria, specifically *L. crispatus*, *L. gasseri*, *L. iners*, and *L. jensenii* (Redondo-Lopez et al., 1990). The remaining healthy women (±20–30%) have a more diverse vaginal microbiome with low concentrations of *Lactobacillus* species. In the microbial communities of these women, strict anaerobic bacteria are predominant from genera such as *Prevotella*, *Dialister*, *Atopobium*, *Gardnerella*, *Megasphaera*, *Peptoniphilus*, *Sneathia*, *Eggerthella*, *Aerococcus*, *Finegoldia*, *Mobiluncus* etc. (Ravel et al., 2011; Drell et al., 2013). Since an inverse relationship exists between the presence of lactobacilli and BV-associated species, non-diverse *Lactobacillus* dominated microbiomes are considered healthy (Razzak et al., 2011). However, it is still a subject of debate how much variation in the vaginal microbiome can be considered within normal boundaries. Whether these women should be considered healthy or asymptomatic for bacterial vaginosis (BV) remains unclear since many of these anaerobic bacteria are after all common causes of BV (Fredricks et al., 2005; Danielsson et al., 2011). In contrast to bacteria, not every woman has vaginal mycobiota. The proportion of asymptomatic women with fungal vaginal communities ranges between 20 and 50%. The mycobiome’s predominant occupant is *C. albicans*, however, non-*albicans Candida* species such as *C. krusei, C. parapsilosis, C. tropicalis, C. glabrata*, as well as species from other genera like *Saccharomyces, Aspergillus, Alternaria*, and *Cladosporium* can also be present (Goldacre et al., 1981; Barousse et al., 2004; Nowakowska et al., 2004; Drell et al., 2013; Underhill and Iliev, 2014).

Since lactobacilli make up the vast majority of the vaginal microbiome, a large amount of the metabolites present in the vaginal niche, of which lactate is most abundant, are produced by these species. Therefore, most recent studies focus on vaginal health mainly or exclusively by studying lactobacilli and their metabolic properties. Lactic acid and the SCFA that can be found in the female genital tract are produced via fermentation of carbohydrates and degradation of amino acids by various microorganisms, as represented in Table 2 (Amabebe and Anumba, 2020). Lactobacilli use glycogen, produced in the vaginal epithelium, during anaerobic glycolysis to produce lactate. The bacteria do not directly metabolize glycogen. A vaginal α-amylase breaks down glycogen first to maltose, maltotriose, maltopentaose and maltodextrins (Spear et al., 2014). These short polymers are then metabolized to pyruvate via glycolysis. Finally, L- or D-lactate dehydrogenase converts pyruvate to L- or D-lactate. Not all *Lactobacillus* species are able to produce both isomers. *L. iners* only has genes coding for L-lactate dehydrogenase in its genome, while *L. crispatus*, *L. gasseri*, and *L. jensenii* have genes encoding both enzymes (Papagianni, 2012; Witkin et al., 2013). *Lactobacillus* species differ in the amount of lactate they produce and even within the same species, metabolic output can vary between strains. *L. crispatus* dominated microbiomes are generally associated with a high lactate content and acidic vaginal pH (Bai et al., 2012). Although lactate is the main fermentation end product of lactobacilli, it is not their only metabolic product. *L. jensenii* is also capable of producing high amounts of acetate and succinate (Amabebe and Anumba, 2020). Most bacteria that are responsible for the production of the SCFA found in the vagina, are BV-associated species. So, it is no surprise that during BV, lactate levels are lowered, while the concentration of SCFA increases (Spiegel et al., 1980; Stanek et al., 1992; Yeoman et al., 2013). A few examples of BV-associated species that produce organic acids are: *Peptococcus* (butyrate and acetate production), *Dialister* (propionate production), *Gardnerella vaginalis* (acetate and succinate production), *Bacteroides* (succinate production), gram-positive cocci, and *Clostridium* (caproate production) (Spiegel et al., 1980; Debruères and Sedallian, 1985; Downes et al., 2003; Chaudry et al., 2004; Aldunate et al., 2015). Because archaea, protists, fungi and viruses are largely outnumbered by bacteria, the majority of SCFA in the vagina are produced by bacteria.

**TABLE 2:** Production of fatty acid metabolites by micro-organisms occurring in the vaginal niche (Yassin and Schaal; Hewitt, 1932; Ng and Hamilton, 1971; Gorbach et al., 1976; Lanigan, 1976; Lambert and Armfield, 1979; Love et al., 1980; Stackebrandt et al., 1982; Ezaki et al., 1983; Lorowitz and Bryant, 1984; Russell and Hino, 1985; Marounek et al., 1989; Miles et al., 1991; Braham and Moncla, 1992; Kawai et al., 1996; Macfarlane and Gibson, 1997; Pollack et al., 1997; Al-Mushrif et al., 2000; Downes et al., 2000, 2002; Kageyama and Benno, 2000; Takahashi et al., 2000; Ezaki et al., 2001; Castan and Enfors, 2002; Inui et al., 2004; Zhou et al., 2004; Romanik et al., 2006; Carlier et al., 2007; Song et al., 2007; Boumba et al., 2008; Ciani et al., 2008; Louis and Flint, 2009; Louis et al., 2010, 2014; Murzyn et al., 2010; Sela et al., 2010; Garland, 2011; Paul et al., 2011; Khan et al., 2012; Prabhu et al., 2012; Stahl et al., 2012; Ze et al., 2012; Byung-Chun et al., 2013; Park et al., 2013; Wodke et al., 2013; Kentner et al., 2014; Othman et al., 2014; Summanen and Finegold, 2015; France et al., 2016; Koh et al., 2016; Wang et al., 2016; Bai et al., 2017; LeBlanc et al., 2017; Louis and Flint, 2017; Martín et al., 2017; Rios-Covian et al., 2017; Sechovcová et al., 2017; Crost et al., 2018; Duan et al., 2018; Franke and Deppenmeier, 2018; Moon et al., 2018; Paek et al., 2018; Che et al., 2019; Chen et al., 2019; Hernandez-Sanabria et al., 2019; Linhares et al., 2019; Luu et al., 2019; Mendling et al., 2019; Offei et al., 2019; Ozato et al., 2019; Wang et al., 2019; Zhao et al., 2019; Amabebe and Anumba, 2020; Lenoir et al., 2020; Usta-Gorgun and Yilmaz-Ersan, 2020; Wongkuna et al., 2020; Nakkarach et al., 2021; Negari et al., 2021).

	**Formate (C1)**	**Acetate (C2)**	**Propionate (C3)**	**L-Lactate (C3)**	**D-Lactate (C3)**	**Butyrate (C4)**	**Isobutyrate (C4)**	**Succinate (C4)**	**Valerate (C5)**	**Isovalerate (C5)**	**Caproate (C6)**	**Caprylate (C8)**	**Caprate (C10)**
*Allisonella*			X			X					X		
*Alloscardovia*												X	
*Anaerococcus*		X		X		X		X					
*Arcanobacterium*		X		X				X					
*Atopobium*	X	X		X									
*Bacteroides*		X	X	X		X	X	X		X			
*Bifidobacterium*	X	X	X	X		X		X					
*Blautia*	X	X		X		X		X					
*Bulleidia*		X	X				X						
*Campylobacter*		X		X									
*Clostridium*		X	X			X							
*Corynebacterium*				X				X					
*Dialister*			X										
*Enterobacter*	X	X		X				X					
*Escherichia*	X	X	X	X		X	X		X	X	X		
*Faecalibacterium*	X			X		X							
					X								
*Fusobacterium*		X	X			X	X		X	X			
*Gardnerella*		X	X	X		X	X	X					
*Gemella*		X		X									
*Haemophilus*	X	X			X			X					
*L. crispatus*		X		X									
*L. gasseri*			X	X									
*L. iners*				X									
*L. jensenii*		X		X				X					
*Megasphaera*		X	X	X		X							
*Mobiluncus*		X	X	X		X	X	X					
*Mollicutes*	X	X	X	X		X	X	X		X			
*Moryella*		X		X									
*Mycoplasma*		X		X									
*Olsenella*	X	X		X									
*Peptococcus*		X	X	X		X				X			
*Peptostreptococcus*		X	X	X		X		X	X				
*Porphyromonas*		X	X			X	X	X		X			
*Prevotella*	X	X	X	X		X	X	X					
*Pseudomonas*		X	X			X	X		X	X			
*Ruminococcus*	X	X		X		X		X					
*S. boulardii*		X									X	X	X
*Shigella*		X											
*Shuttleworthia*		X		X		X							
*Solobacterium*		X		X		X							
*Staphylococcus*		X	X	X		X	X	X		X			
*Streptococcus*	X	X		X									
*Veillonella*		X	X										

**Only published results are depicted as an X. For the other metabolites or organisms, no data is available. Organisms that were reported to inhabit the vaginal niche, yet for which no fatty acid metabolite production has been described, are depicted in Supplementary Table 1.**

**FIGURE 1:**
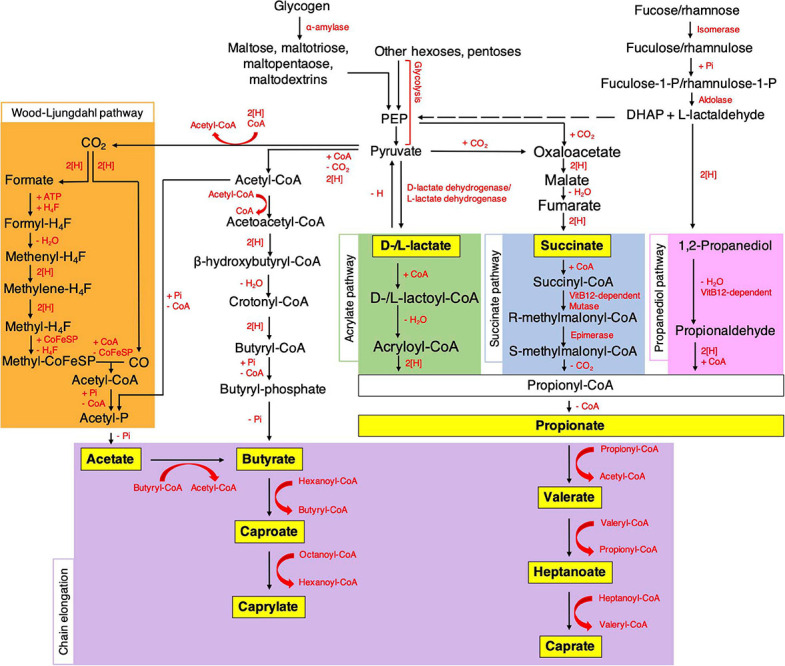
Microbial pathways for the biosynthesis of SCFA and MCFA in the gut. [H] indicates redox reactions which involve electron carriers. PEP, phosphoenolpyruvate; DHAP, dihydroxyacetonephosphate; CoA, coenzyme A; H4F, tetrahydrofolate; Pi, inorganic phosphate; CoFeSP, corrinoid iron sulfur protein; ATP, adenosine triphosphate. Details are given in the main text (Koh et al., 2016; O-Thong et al., 2020).

There is a lot of information available on the metabolic pathways of vaginal lactobacilli. The biosynthetic pathways by which other vaginal bacteria produce SCFA are less well documented. In the gut on the other hand, these pathways are well understood and described in detail (Koh et al., 2016). Figure 1 represents a schematic overview of fatty acid metabolism. Acetate can be produced from pyruvate directly via acetyl-CoA or via the branched Wood-Ljungdahl pathway. In the methyl branch, CO_2_ is reduced to formate to eventually produce a methyl group. In the carbonyl branch, CO_2_ is reduced to carbon monoxide, which is then combined with the bound methyl group and coenzyme A to form acetyl-CoA (Ragsdale and Pierce, 2008). Succinate can be formed by reversing some reactions of the tricarboxylic acid cycle. Pyruvate is first converted to oxaloacetate by carboxylation. Oxaloacetate is then reduced to malate, fumarate and eventually succinate (Connors et al., 2018). Propionate can be synthesized via three different pathways: the acrylate pathway, the succinate pathway and the propanediol pathway. In the acrylate pathway, lactate is first combined with coenzyme A to form lactoyl-CoA followed by a dehydration and reduction to produce propionyl-CoA. In the succinate pathway, succinate is converted to methylmalonyl-CoA, which is then decarboxylated to yield propionyl-CoA. In the propanediol pathway, deoxyhexose sugars fucose and rhamnose are degraded to form 1,2-propanediol, followed by a dehydration and addition of coenzyme A to produce propionyl-CoA. Via dihydroxyacetone-phosphate and methylglyoxal, which are glycolysis intermediates, 1,2-propanediol can also be produced from other sugars. The final step of all three pathways is to omit coenzyme A to form propionate (Hetzel et al., 2003; Scott et al., 2006; Louis and Flint, 2017). Butyrate can be formed by the condensation reaction of two molecules of acetyl-CoA, followed by reduction to butyryl-CoA, which is then converted to butyrate (Louis et al., 2004; Louis and Flint, 2017). In the vagina, these pathways could possibly be different since there are differences between the characteristics of both niches like the pH, presence of certain sugars, acids, enzymes, oxygen availability, etc. (Fallingborg, 1999; Hill et al., 2005; Krauss-Silva et al., 2014; Zheng et al., 2015). Very little research is performed on the vaginal mycobiome and its metabolites and thus, no conclusions can be made at this point. Additionally, it was established before that fungal species also produce significant amounts of SCFA, but whether they are able to produce them in the vaginal niche and their production pathways need to be further investigated (Pinu et al., 2018). Research shows that metabolome deviations can be correlated to vaginal dysbiosis, such as BV and VVC, however, many questions regarding the role of these metabolites in health and the potential of using them in therapy, remain unanswered (Vitali et al., 2015; Tachedjian et al., 2017).

#### From Gut to Vagina: Transmission of Microbes and Their Metabolites

The microbiome of the gut is a complex and heterogeneous ecosystem that consists of more than 1,100 species (Scarpellini et al., 2015). A healthy eubiotic gut is dominated by *Firmicutes* and *Bacteroidetes* but deficient in *Proteobacteria* species that promote inflammation (Cammarota et al., 2015). *Firmicutes* and *Bacteroidetes* make up more than 90% of all the gut microbiota. The predominant genera are *Bacteroides*, *Bifidobacterium*, *Eubacterium*, *Clostridium*, *Peptococcus*, *Peptostreptococcus*, and *Ruminococcus* (Salminen et al., 1998; Guarner and Malagelada, 2003). The most abundant bacterial species in the gut of healthy adults is *Faecalibacterium prausnitzii* of the *Firmicutes* phylum. It represents more than 5% of the total intestinal bacterial population (Hold et al., 2003; Miquel et al., 2013). *Firmicutes* species are the main butyrate producers, while *Bacteroidetes* species the main acetate and propionate producers in the gut (Yang et al., 2013; Salonen et al., 2014; Vital et al., 2014; Aguirre et al., 2016). Archaea, protists, fungi and viruses can also be found in the gut, but to date their exact composition is not completely resolved (Nash et al., 2017; Nieves-Ramírez et al., 2018; Shkoporov et al., 2019; Kim et al., 2020). The mycobiome of the human gut is low in diversity compared to bacteria with *Saccharomyces*, *Malassezia*, and *Candida* as predominant genera (Nash et al., 2017).

Crosstalk and transfer between microorganisms of the vagina and gut is possible both horizontally, from gut to vagina within the same individual, and vertically, from mother to child. Vaginal microorganisms including the dominant *Lactobacillus* species are believed to originate from the gut (El Aila et al., 2009a, b, 2011; Amabebe and Anumba, 2018). Species of the phyla *Firmicutes*, *Bacteroidetes*, *Proteobacteria*, *Actinobacteria*, and *Fusobacteria* are found in both niches (Eckburg et al., 2005; Kaur et al., 2020). In one study, vaginal and rectal swabs were collected from 132 pregnant women (35–37 weeks of gestation) (El Aila et al., 2009b, 2011). In 36% of these women the same bacterial species were identified in their vagina and rectum, of which 68% of the isolated species showed identical random amplified polymorphic DNA (RAPD) patterns, indicating substantial genotypic similarity. This microbial transfer from gut to vagina is striking for recurrent BV. Women with high concentrations of BV-associated bacteria in their rectum are prone to recurrent BV caused by repeated re-infection from rectum to vagina (Marrazzo et al., 2012). Vertical transmission of microorganisms depends on the method of delivery. Newborns delivered vaginally have microbial communities in their gut that are similar to the microbiota of the mother’s vagina. Their intestinal microbiome is dominated by *Lactobacillus*, *Prevotella*, *Sneathia*, and *Bifidobacterium* species (Dominguez-Bello et al., 2010). Bacteria of other genera like *Faecalibacterium*, *Roseburia*, *Staphylococcus*, *Streptococcus*, *Atopobium*, *Akkermansia*, *Escherichia*, *Bacteroides*, *Methanobrevibacter*, *Peptostreptococcus*, and *Veillonella* can also be present (Grölund et al., 1999; Biasucci et al., 2008; Pantoja-Feliciano et al., 2013; Mueller et al., 2015; Perez-Muñoz et al., 2017). Newborns delivered by C-section have intestinal microbiota that originate from the environment and the microbiota of the skin of the mother. The microbiome of their gut shows less diversity and a lower microbial richness (Dominguez-Bello et al., 2010; Azad et al., 2013). It is dominated by *Staphylococcus*, *Corynebacterium* and *Propionibacterium* species while *Bifidobacterium* species are absent (Dominguez-Bello et al., 2010; Perez-Muñoz et al., 2017). Additionally, there is increasing evidence that links intestinal microbiota to postnatal development of the immune system (Cho and Norman, 2013; Kristensen and Henriksen, 2016). Since the composition of the intestinal microbiome differs depending on the method of delivery, this could have an effect on the development of the immune system (Dominguez-Bello et al., 2010). It is already confirmed that children born by C-section have an increased risk of immune system disorders such as bronchiolitis, gastroenteritis, inflammatory bowel disease, leukemia and allergies such as asthma, hay fever and eczema (Cho and Norman, 2013).

Since transfer of microbes happens, the SCFA will move along with their producers. Acetate, propionate and butyrate are the major SCFA present in a healthy gut. Succinate and lactate are generally detected in lower concentrations. The amount of SCFA differs along the length of the gut. The total SCFA concentration is the highest in the caecum (131 mmol/kg intestinal content) followed by the ascending (123 mmol/kg), transverse (117 mmol/kg) and descending colon (80 mmol/kg) (Cummings et al., 1987). The availability of substrates and free water is the highest in the caecum making it the primary site of fermentation (Chakraborti, 2015). SCFA have important functions in the gut. They promote mucus production, stimulate the production of antimicrobial peptides, increase the expression of intestinal tight junction proteins, maintain the integrity of the intestinal epithelial barrier and serve as important energy substrates for colonocytes (Roediger, 1982; Willemsen et al., 2003; Ewaschuk et al., 2008; Peng et al., 2009; Zhao et al., 2018). In contrast to the gut, the predominant organic acid in a healthy vagina is lactic acid (±120 mM) (Al-Mushrif et al., 2000; Gajer et al., 2012). The concentrations of acetic (0–4 mM), propionic (<1 mM), butyric (<1 mM), and succinic acid (<1 mM) during eubiosis are much lower (Al-Mushrif et al., 2000; Chaudry et al., 2004; Mirmonsef et al., 2011; Mirmonsef et al., 2012; O’Hanlon et al., 2013). During BV, these proportions change. The concentration of lactate drops below 20 mM and the SCFA are increased: acetate (< 120 mM), propionate (2–4 mM), butyrate (2–4 mM), succinate (<20 mM) (Al-Mushrif et al., 2000; Chaudry et al., 2004; Mirmonsef et al., 2011, 2012; Gajer et al., 2012). The functions of lactate and SCFA in the vagina are discussed further. Transfer of microbes between gut and vagina is possible so one would think the microbial composition of both would be similar. However, due to differing characteristics of the niches different species thrive and become dominant in the microbiome. This makes that their metabolites lactate, acetate, butyrate, succinate and propionate are present in both niches but in completely different concentrations.

#### Fatty Acid Metabolites Originating From the Vaginal Mucosa

In addition to production by vaginal microorganisms, lactate can also be produced by the vaginal mucosa itself (Weinstein et al., 1936; Weinstein and Howard, 1939; Linhares et al., 2011). Epithelial cells have access to limited oxygen, glucose and other essential nutrients that diffuse from underlying tissues. Therefore, the dominant metabolism in the vaginal mucosa is the anaerobic fermentation of glucose (Baron and Merk, 2001). Glycogen, which is stored in vaginal epithelial cells, is first converted to glucose and then metabolized to pyruvate and adenosine triphosphate via glycolysis. Eventually, pyruvate is converted to lactate (Gross, 1961). These epithelial lactate molecules diffuse to the vaginal lumen and maintain the acidic pH of the vagina together with lactate produced by vaginal bacteria. Most epithelial lactate is probably produced in the intermediate vaginal epithelium cell layer, because glycogen metabolism is highest here (Linhares et al., 2011). It is not clear yet whether the primary source of vaginal lactate is the vaginal mucosa or vaginal microbiota and whether this is the same in all women. Boskey, Cone (Boskey et al., 2001) concluded that vaginal bacteria are the primary source of lactate in the vagina, because they found that more than 50% of the lactate in the vaginal samples of most participants of their study was D-lactate. Epithelial cells can only produce L-lactate while bacteria can produce both L- and D-lactate (Brin, 1965; Smith et al., 1989; Bongaerts et al., 1997; McCabe et al., 1998). However, the percentage of D-lactate in the vaginal samples ranged between 6 and 75%, which is too broad of a range to conclude that bacteria are the dominant source in all women (Boskey et al., 2001). Also, the concentration of lactobacilli is uniform throughout the whole vagina and yet the pH in the lower part of the vagina is more acidic compared to the middle and upper part (Chen C. et al., 2017; Lykke et al., 2021). This suggests a rather important role of the vaginal mucosa in the determination of the vaginal pH. To the best of our knowledge, evidence that the vaginal epithelial cells produce significant amounts of SCFA remains absent.

### Functions of Fatty Acid Metabolites in the Healthy Vagina

#### Fatty Acid Metabolites Influence the Local pH

**FIGURE 2:**
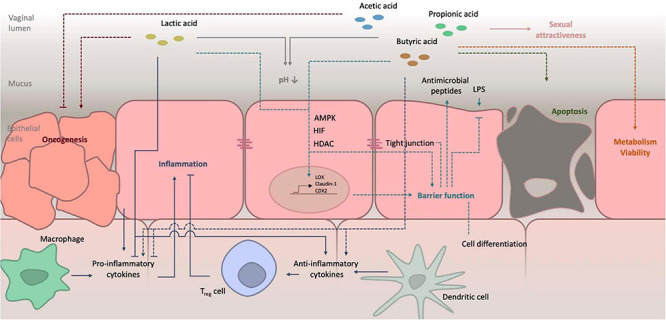
Schematic representation of the effects of lactate and SCFA on host metabolism. Details on their role in pH balance, barrier function and inflammation are schematized. Further details as well as references are given in the main text. LPS, lipopolysaccharide; AMPK, AMP-activated protein kinase; HIF, hypoxia-inducible factor; HDAC, histon deacetylase.

The average healthy pH of the vagina is found to be around 4 ± 0.5 although some studies report even lower pH values of 2.8–4.2 (O’Hanlon et al., 2013). Variations in the vaginal pH exist and are caused by recent sexual activity, condom use, hormonal activity or treatment, age, the menstrual cycle and various types of illnesses and infections. The pH also alters across different geographical locations and ethnicity. The low pH of the human vagina is quite unique if you compare it to other mammals, in which the vaginal pH ranges from 5.4 to 7.8 (Miller et al., 2016). The main reason for this high acidity in humans, is likely the dominance of lactobacilli (Boskey et al., 2001). It has been established that more than 70% of the bacteria in the human vagina are lactobacilli, while in other mammals this only accounts for 1%. Under the influence of high estrogen levels, glycogen is deposited in the vaginal epithelial cells, mainly in the intermediate layers (Ayre, 1951; Gross, 1961). The metabolism of glycogen accounts for energy production, invested in proliferation and maturation of epithelial cells. Breakdown of glycogen by human α-amylases, leads to smaller polymers, such as maltose, maltotriose, and α-dextrines (Amabebe and Anumba, 2018). In anaerobic conditions, such as those in the vaginal cavity, lactobacilli convert these first to pyruvate and later to lactate by activity of lactate dehydrogenase. Lactate in its turn lowers the local pH. Although the resident bacteria were shown to be the main source of lactate, producing both D- and L-isomers, also epithelial cells can breakdown some glycogen into lactic acid, however, only producing the L-isomer (Witkin et al., 2013). The roles of both isomers are also slightly different. It has been suggested that the protective qualities of D-lactate against particular infectious agents are higher compared to those of the L-isomer, although this has been countered by others (McWilliam Leitch and Stewart, 2002; Aldunate et al., 2013; Witkin et al., 2013). The difference in quantity of both isomers might also partially explain the difference in protecting qualities between different *Lactobacillus* species, as they produce different amounts of both (Boskey et al., 2001). In humans, the vaginal pH inversely correlates with the amount of lactate present. This implies that lactate is the main cause of acidification in this niche (O’Hanlon et al., 2013). Figure 2 depicts a schematic representation of the effects of lactate and SCFA on metabolism, including their effect on the local pH. Lactic acid is a weak acid, indicating that it only partially dissociates in water to form lactate and a proton (H^+^). Its pKa is 3.89, so below a pH of about 3.9, lactic acid exists in its protonated state, while above this pH the lactate anion predominates (Tachedjian et al., 2017). Therefore, because the average pH in the vaginal niche is about 4, in this review we will refer to this molecule as lactate, even though the pH can drop below 3.89 and lactic acid will be present (O’Hanlon et al., 2013). This protonated acid is membrane-permeable and can thus diffuse into microbial cells. Upon entering, the cytosol is acidified, which leads to impaired cellular functioning, membrane permeabilization and cell death (Alakomi et al., 2000). In contrast, in the deprotonated form, lactate has no antimicrobial activities. It is thus of importance that the pH of the vagina is sufficiently acidic to allow lactic acid to exist and exert its protective qualities.

Apart from lactic acid, also some other organic acids occur in the vaginal niche, such as acetic acid, propionic acid, butyric acid and succinic acid. These are generally weaker acids compared to lactic acid, with pKa values of 4.76, 4.82, 4.87, and 4.16–5.61, respectively. This indicates that they exist in their protonated form at pH levels lower than 4.5 and will thus more readily penetrate and acidify pathogenic bacteria in comparison to lactic acid. However, the amounts in which they are observed in the vaginal cavity and thus contribute to the local pH are negligible (Tachedjian et al., 2017). During periods of bacterial vaginosis, the pH of the vagina typically increases, as well as the levels of some SCFA (Yeoman et al., 2013; Hoffman et al., 2017). Measurement of acetic acid levels in the headspace of vaginal fluid samples, has even been suggested as a diagnostic tool for bacterial vaginosis (Chaudry et al., 2004).

#### Fatty Acid Metabolites Affect the Epithelial Barrier Function

The vaginal mucosa of healthy women is composed of several parts. The first line of defense against pathogens is the mucus layer covering the epithelial cells (Lacroix et al., 2020). This cervicovaginal mucus consists for more than 95% of water. Mucins are glycosylated proteins produced by epithelial cells. After polymerization, the protein chains are secreted into the vaginal lumen, where they bind water and form hydrogels. Mucins cause the mucus to become elastic and viscous. Apart from mucins and water, vaginal mucus also contains other proteins, nucleic acids, fatty acids and cells. The mucus layer covering the epithelial cells performs several functions. Apart from safeguarding fertility, its main role is to protect the vagina and uterus from harmful micro-organisms. The latter function comprises both the provision of a favorable environment for the healthy constituents of the microbiota as well as creating an unfavorable niche for pathogens, by accumulating immune modulatory components and limiting pathogen diffusion (Yarbrough et al., 2015). The outer-most cellular section of the vagina consists of multiple layers of stratified squamous epithelium, which rest on a basal cell layer or lamina propria. The uterus as well as the upper part of the cervix are lined with columnar epithelium. The epithelial cells are surrounded by tight junctions which close off the intercellular space, thereby disconnecting the exterior or apical surrounding from the deeper, basolateral, tissues. Together, the mucus and epithelial cells can be termed the epithelial barrier, as they protect the host from pathogens encountered in the exterior environment, a function vital for maintaining proper homeostasis within the vagina. When the structure and composition of the mucus layer are aberrant, infections are likely to arise (Goldenberg et al., 2000). During pregnancy, this can be linked to preterm birth (Smith-Dupont et al., 2017). The tightness of the barrier as composed by *in vitro* cultured epithelial cells, can be quantified using transepithelial electrical resistance (TEER) (Gorodeski, 1996; Srinivasan B. et al., 2015). The effect of several parameters, such as age, hormonal levels, mental status, composition of the microbiome and vaginal lubricants on barrier function has been investigated, using this technique (Gorodeski, 2001a, b, 2007; Li et al., 2015; Tsata et al., 2016; Ayehunie et al., 2018).

Only little is known about the effect of different short or medium chain fatty acids on the epithelial barrier function of the vagina. It is, therefore, of interest to take a look at the interplay between both factors in the gut, where much more knowledge has been acquired over the past years. It has been shown many years ago, that SCFA have a beneficial role in enforcing the epithelial barrier function in the gut, although the exact mode-of-action is still not fully understood (Harig et al., 1989). It is observed by various research groups that SCFA, such as acetate, propionate and butyrate, strengthen the gut epithelial barrier function by stimulating tight junction formation and prevention of damage, for instance imposed by lipopolysaccharides (Peng et al., 2009; Hsieh et al., 2015; Feng et al., 2018). The effect of butyrate is most thoroughly investigated. A myriad of mechanisms explaining the altered formation of tight junctions has been suggested. It was already established that butyrate inhibits histone deacetylases (HDAC), thereby altering gene expression (Della Ragione et al., 2001). Ohata et al. (2005) show that in this manner, expression of the LOX gene encoding lipoxygenase was increased upon addition of butyrate or propionate to intestinal cells. This enzyme produces hydroxy derivatives of arachidonic acid, which were shown to decrease permeability of tight junctions. In another study, the effect of butyrate on expression of the Claudin-1 gene, which encodes a major component of tight junctions, was shown and ascribed to the increased association between the transcription factor SP1 and the Claudin-1 promoter (Wang et al., 2012). On the other side, butyrate was shown to repress the expression of the Claudin-2 gene, which encodes a tight junction protein that promotes permeability, through the IL-10 receptor α subunit (Zheng et al., 2017). Yet another study demonstrates the involvement of the AMP-activated protein kinase (AMPK) pathway in the butyrate-regulated increase of tight junction assembly (Peng et al., 2009). The AMPK pathway activates expression of the CDX2 transcription factor by histone modification, which increases epithelial cell differentiation and thereby barrier function (Sun et al., 2017). Alternatively, the effect of AMPK on epithelial barrier function might be caused by a decreased phosphorylation of the myosin II regulatory light chain (MLC2) and increased phosphorylation of protein kinase C β2 (PKCβ2) (Miao et al., 2016). Butyrate and other SCFA have also been shown to increase oxygen consumption by intestinal cells (Kelly et al., 2015). This causes stabilization of the hypoxia-inducible factor, which, by activating a plethora of targets genes, reinforces the barrier function (Pasolli et al., 2017). Recently, butyrate exposure and improved tight junction integrity were shown to be linked through the increased expression of the actin-binding protein synaptopodin (Wang et al., 2020). The induction of this epithelial barrier promoting protein is possibly linked to inhibition of HDAC. This beneficial effect of butyrate on TEER does not seem to be universal. In a recent *in vitro* setup, Vancamelbeke and co-workers could confirm the increased TEER of human primary colonic monolayers upon butyrate exposure. However, after induction of inflammation, butyrate reduced epithelial barrier integrity. Moreover, presence of butyrate at higher concentrations has been shown to induce toxicity to the epithelial cells, due to activation of apoptosis (Peng et al., 2007; Liu et al., 2018). This balance between the beneficial and harmful effect must thus be considered for application.

The role of lactate in maintenance or reinforcement of both the vaginal and intestinal epithelial barrier function is severely understudied. Okada et al. (2013) showed that feeding mice lactate after a period of starvation enhances enterocyte proliferation, thereby contributing to the barrier function of the gut. Similar to butyrate and other SCFA, lactate can inhibit HDACs, although the concentrations necessary to achieve this are higher than what is present in the gut (Latham et al., 2012; Schilderink et al., 2013). In the vaginal niche, the concentration of lactate is higher and potentially sufficient to inhibit HDAC activity. However, lactate is a less potent inhibitor. The polar hydroxyl moiety that is present on the second lactate carbon may reduce binding to the HDAC active site, which is lined by hydrophobic residues, and explain this lower activity (Vannini et al., 2004). Although the exact role of both lactic acid enantiomers is often not investigated, in this particular case, different activities were observed for both, with D-lactate (10 mM) being more potent than L-lactate (40 mM) (Latham et al., 2012). Nevertheless, at physiological vaginal concentrations, HDAC inhibition was demonstrated *in vitro* (Wagner et al., 2015).

Whether and how lactate might potentiate the vaginal epithelial barrier function is thus not evident.

#### Fatty Acid Metabolites Influence the Local Immune Response

Several organic acids present in the human body have immunomodulatory functions. An inflammatory response is necessary to prevent invasion and infection by pathogens. However, chronic inflammation is characterized by high levels of pro-inflammatory cytokines/chemokines and potentially damages the tissue which, in its turn, can lead to hypersusceptibility for other pathogens, such as HIV (Couzin-Frankel, 2010; Passmore et al., 2016). Many of the symptoms of vaginal candidiasis are caused by the inflammation secondary to the infection (Cassone, 2015). Certain organic acids can induce or repress the local immune response. At the intestinal mucosa, these anti-inflammatory functions are widely studied and several modes-of-action have been identified. Inhibition of HDACs, promotion of histone acetylation that consequently affects genes involved in the inflammatory response, inhibition of LPS-induced NF-kB signaling and induction of anti-inflammatory cytokines, such as IL-10 (Segain et al., 2000; Cox et al., 2009; Chang et al., 2014). Particularly in the gut, it was concluded that the presence of SCFA producing microbes shows an advantage and even a potential therapeutic strategy for diseases like ulcerative colitis and Crohn’s disease (Parada Venegas et al., 2019). Lactate also protects the gut from inflammation-mediated damage. It has been observed that lactate-pretreatment of a murine 2,4,6-trinitrobenzenesulfonic acid induced colitis model prevented intestinal inflammation (Iraporda et al., 2016). However, it has also been shown that certain organic acids can activate the immune system when present at higher concentrations (Vinolo et al., 2009, 2011). The situation in the vaginal niche is less well-studied. First, lactic acid, both protonated L- and D-isomers, elicits an anti-inflammatory response as it dampens an overactive immune response. Based on studies in which transmission of HIV was investigated, it can be assumed that both enantiomers perform similarly when it comes to modulation of the local immune response (Hearps et al., 2017). Lactic acid stimulates production of anti-inflammatory cytokines/chemokines, such as IL-1 receptor antagonist, and inhibits Toll-like receptor-induced production of pro-inflammatory actors, such as IL-6 and IL-8, by cervicovaginal epithelial cells (Hearps et al., 2017; Tyssen et al., 2018; Delgado-Diaz et al., 2019). Lactic acid thereby likely plays a role in protection of the unborn fetus and protection of the pregnancy against inflammation as well as providing protection against HIV. However, using the same mechanisms, lactic acid also promotes oncogenesis (Witkin, 2018). Presence of certain SCFA, such as acetic acid, propionic acid and butyric acid, as well as succinic acid at low pH and at concentrations that characterize eubiosis, do not interfere with the effect of lactic acid on the local immune response (Delgado-Diaz et al., 2019). However, prolonged treatment of cervicovaginal cells with these SCFA in higher concentrations typical for dysbiosis and higher pH, elicits conflicting effects. While production of the pro-inflammatory cytokines TNFα and IL-1β are upregulated, production of IL-6, IL-8, and others were downregulated (Mirmonsef et al., 2012; Delgado-Diaz et al., 2019). The net effect of these changes on the immune status in the vaginal niche remains unknown. Moreover, several research groups report seemingly contradictory results on the subject (Mirmonsef et al., 2012; Aldunate et al., 2015; Delgado-Diaz et al., 2019). This conflicting data may result from differences in the cell type or method used for stimulation of the cells, e.g., using Toll-like receptor agonists PAM, imiquimod, PIC or lipopolysaccharides (Delgado-Diaz et al., 2019).

#### SCFA Play a Role in Sexual Attractiveness

Although rather controversial, SCFA present in the vaginal fluid were also suggested to play a role in sexual attractiveness of women (Michael and Keverne, 1970; Michael et al., 1974, 1975). The composition of the SCFA pool, also called copulins in this respect, varies with the stage in the menstrual cycle (Michael et al., 1975). It has been shown that these molecules act as pheromones that affect the attractiveness as rated by men and the women themselves (Grammer et al., 2005; Williams and Jacobson, 2016).

#### Fatty Acid Metabolites Serve an Antimicrobial Function

It has been shown on multiple occasions, mainly in the gut, that SCFA and lactic acid possess antimicrobial activity. It can generally be accepted that this antimicrobial activity is mediated mainly by lowering intracellular pH and concomitant disturbance of cellular metabolism. To elicit this effect on the intracellular environment, these acids should be available in their protonated and thus membrane-permeable form. Once inside the cell, the acids dissociate, thereby leading to an increase in protons and anions. An important prerequisite is that the pH of the local niche should thus be below the pKa of the acid. The exact mechanism by which the acids affect virulence of the pathogenic organisms is not known, although several options have been discussed. The effect of SCFA against intestinal pathogens, such as *Salmonella* species, *E. coli* and *Campilobacter jejuni*, has been investigated. SCFA modulate growth, motility, biofilm formation and quorum sensing by these organisms (Nakamura et al., 2009; Amrutha et al., 2017; Jacobson et al., 2018; Lamas et al., 2019). Remarkably, it has also been shown that subinhibitory concentrations of these SCFA can alter gene expression of pathogenic bacteria in such a way that virulence is favored (Lamas et al., 2019). It must be noted, however, that this effect is strongly dependent on the type of pathogen.

*Lactobacillus*-based probiotics are well known for their gut microbiome modulatory functions, where they are used against diarrhea and other gastrointestinal disorders (Azad et al., 2018). Part of the anti-infection activity of these bacterial strains is attributed to their production of lactic acid and consequent lowering of the intracellular pH (Fayol-Messaoudi et al., 2005). Most research specific to the vaginal niche has been devoted to the antimicrobial effect of lactic acid. It has been shown that lactic acid produced by lactobacilli can inhibit both bacteria associated with BV, such as *Gardnerella vaginalis*, *Atopobium vaginae* and *Clostridium perfringens*, and viruses, such as HIV and HSV-2 (Conti et al., 2009; O’Hanlon et al., 2011; Aldunate et al., 2013; Amin et al., 2017). Remarkably, there is no harmful effect against natural inhabitants of the vagina, such as *L. jensenii* and *L. crispatus* (O’Hanlon et al., 2011). Important to mention, however, is that these effects are strongly pH dependent (pKa of lactic acid is 3.89) as only the protonated form of the acid has these antimicrobial properties. It was indeed shown that at slightly higher pH, lactate does not seem to exhibit similar antiviral nor antibacterial activity (Lai et al., 2009; O’Hanlon et al., 2011). Furthermore, it should be kept in mind that the vaginal pH fluctuates, for example during intercourse, when semen is deposited in the vagina, the pH rises to neutral, indicating that the therapeutic potential of lactic acid should be reconsidered (Fox et al., 1973). It is, however, likely that lactic acid can also affect pathogens in other ways apart from disturbing the intracellular pH balance. It has been suggested that it can also liberate lipopolysaccharides from the outer membranes of bacterial cells and potentially denature viral proteins (Alakomi et al., 2000; Aldunate et al., 2013, 2015). The difference between L- and D-lactic acid in terms of antibacterial and antiviral activity has been studied, although not extensively. It has been reported that L-lactic acid is much more active against particular bacterial pathogens, such as *E. coli*, as well as viral pathogens, such as HIV (McWilliam Leitch and Stewart, 2002; Aldunate et al., 2013). Compared to lactic acid, the SCFA seem to be less potent in targeting pathogenic micro-organisms. Despite the higher pKa of acetic acid (4.76), which will thus be present in the vagina in its protonated form more readily compared to lactic acid, its antimicrobial activity was not found to be as strong as that of lactic acid, potentially proving the importance of the multifactorial activity of lactic acid (Aldunate et al., 2015).

#### Other Functions of Fatty Acid Metabolites

SCFA function as an energy source to host cells, accounting for about 10% of the daily caloric need (Bergman, 1990). They act as substrates for metabolism of glucose, lipids and sterols (LeBlanc et al., 2017). In the gut, mainly butyrate is metabolized by colonocytes, whereas other absorbed SCFA end up in the blood stream, where they provide energy to peripheral tissues (Koh et al., 2016; Amabebe and Anumba, 2020). These molecules have also been shown to reduce diarrhea as they inhibit loss of fluids and electrolytes and stimulate uptake of sodium and chloride (Binder and Mehta, 1989; Rabbani et al., 1999; Binder, 2010). Apart from the potential role of SCFA as antimicrobial actors, they have also been shown to induce expression of antimicrobial peptides by intestinal cells (Zhao et al., 2018). SCFA have also been shown to protect against carcinogenesis in the intestinal system, whereas lactic acid stimulates survival of malignant cells (Hinnebusch et al., 2002; Witkin, 2018). As far as we know, the roles of SCFA in energy metabolism, electrolyte recovery, antimicrobial peptide expression and cancer progression in vaginal epithelial cells, have not yet been investigated. It is also noteworthy that there is a significant difference between host-directed toxicity of L- and D-lactic acid. While L-lactic acid is produced by epithelial cells and is a harmless component of human cell metabolism, exposure to high levels of D-lactic acid is dangerous (Pohanka, 2020). Production of the D-enantiomer is derived from microbial growth. Under normal circumstances, it is present in the blood in low concentrations, not toxic to the host. In specific situations, however, such as short bowel syndrome or other intestinal malfunctioning, D-lactic acid is overproduced, leading to D-lactic acidosis (Kowlgi and Chhabra, 2015). In such conditions, the compound can be neurotoxic (Thurn et al., 1985; Munakata et al., 2010).

## The Role of Fatty Acid Metabolites During Vaginal *Candida* Infections

### Vaginal *Candida* Infections

Worldwide, 70–75% of females suffer from VVC at least once during their life. Approximately 50% of the initially infected women experience a second episode of VVC, while 5–10% face at least four episodes each year, indicating recurrent vulvovaginal candidiasis or RVVC (Sobel, 2007; Gonçalves et al., 2016). However, the actual frequency with which these infections are reported is still underestimated (Parolin et al., 2015). VVC and especially RVVC severely impair the wellbeing, and quality of life; also, they are typically associated with mental distress, low self-esteem, physical pain and sexual dysfunction. In addition, sporadic reports indicate an involvement in late miscarriage, preterm labor, infertility and pelvic inflammatory disease (Gonçalves et al., 2016). Apart from the mental and medical discomfort, these infections impose a substantial financial cost to the patients and society for diagnosis and treatment, reaching up to 1 billion dollars yearly in the US (Zhou et al., 2009). Additionally, research on female specific conditions is underrepresented and underfunded compared to males (Holdcroft, 2007a, b).

*Candida albicans* and *Candida glabrata* are the two species most-frequently isolated from women suffering from VVC, with frequencies of 80 and 2–5%, respectively (Gonçalves et al., 2016). In addition, both organisms may increase each other’s virulence in the female reproductive tract. Major risk factors for VVC are the disturbance of the local microbiome by antibiotic therapy, immunosuppression or host-related factors such as pregnancy and uncontrolled diabetes mellitus. Certain behavioral risk factors include the use of oral contraceptives, poor hygiene and restrictive clothing (Donders et al., 2010). The exact cause of RVVC is unknown, though, it has been suggested to be a hypersensitivity disorder associated with allergic rhinitis and allergic skin disorders (Guo et al., 2012).

The current treatment recommendations of uncomplicated VVC by the American Centers for Disease Control and Prevention (CDC) are topical formulations or a single oral dose of fluconazole (Pappas et al., 2016). For more severe, acute VVC, multiple doses of fluconazole are administered. To treat a *C. glabrata* infection, topical therapy is combined with amphotericin B. RVVC is treated by daily oral administration of fluconazole, after which therapy is continued weekly for 6 months. It is noteworthy, however, that 40–50% of women treated for RVVC will experience recolonization with *Candida* within 30 days after therapy cessation (Sobel et al., 2001). Furthermore, after prolonged treatment, resistance can occur (Marchaim et al., 2012). In Europe, the International Union against Sexually Transmitted Infections (IUSTI) guidelines describe treatment of RVVC according to the ReCiDiF protocol (for Recurrent *Candida* infections treated with Degressive individualized doses of Fluconazole), where personalized reduction of the azole treatment is vital (Donders et al., 2008; Sherrard et al., 2011; Donders and Viera Baptista, 2018). This protocol allows to find the lowest drug dose to remain symptom-free and states that some women do not respond to therapy due to a shift toward azole resistant non-*albicans Candida* species.

### Correlation Between the Vaginal Metabolome and Risk of VVC

As mentioned before, the vaginal microbiota is the prime producer of vaginal metabolites. When the microbiome and consequently the metabolome fluctuates, the chance arises for pathogenic bacteria and fungi to proliferate, dominate and cause infections. Several studies have investigated the microbiome-metabolome fluctuations upon bacterial vaginosis, and associate BV with changes in metabolome composition, such as increased levels of the SCFA acetate and succinate (Srinivasan S. et al., 2015; Vitali et al., 2015). However, little is known about fluctuations in the microbiome and metabolome upon VVC. Ceccarani et al. (2019) analyzed the vaginal microbiome and metabolome upon VVC for the first time. Compared to healthy subjects, increased levels of *Gardnerella*, *Faecalibacterium*, and *Prevotella* were observed. Furthermore, they established that decreased levels of *L. crispatus* correlated with increased levels of *L. iners* and *L. gasseri* in VVC-associated vaginas. In addition, the comparison of the metabolic profiles revealed a unique fingerprint of VVC-affected women. Vaginal fluids of VVC-affected patients were enriched in trimethylamine N-oxide (TMAO), taurine, methanol, isopropanol, and O-acetylcholine, and showed lower concentrations of lactate, 4-hydroxyphenylacetate, phenylalanine, pi-methyl histidine, and glycine (Ceccarani et al., 2019). However, it remains unknown whether these changes in metabolome composition are the cause of the VVC infection or are merely present as a result of the infection.

### The Effect of Fatty Acid Metabolites on *Candida* Growth and Virulence

**FIGURE 3:**
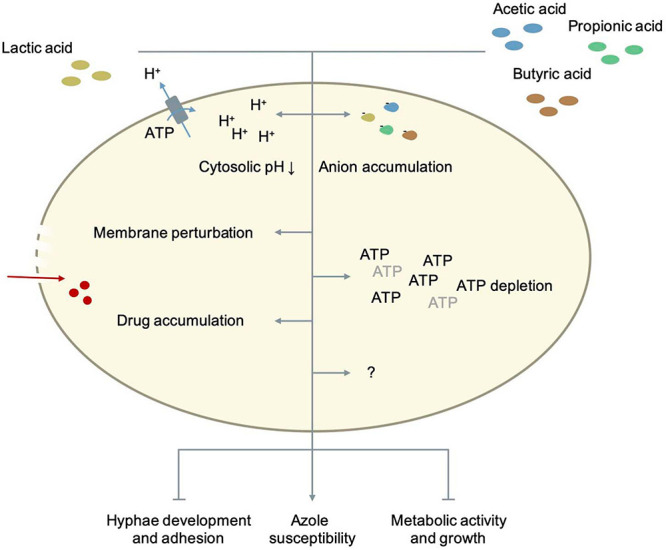
The role of fatty acid metabolites in *Candida* metabolism and pathogenesis. Details as well as references are given in the main text.

Pathogenic *Candida* species can alter their central carbon metabolism to utilize lesser-preferred carbon sources like SCFA instead of glucose. This metabolic versatility is important for their virulence in niches like the vaginal tract that are often low in or deprived of glucose (Childers et al., 2016). The entry of lactic acid and SCFA like acetic acid in the fungal cells is expected to happen mainly via passive diffusion. The vaginal pH is close to or below the pKa of the weak acids (pKa_acetic acid_ = 4.76; pKa_lactic acid_ = 3.89) so that they mostly occur in undissociated form (Lourenco et al., 2018). Although in *C. albicans* and *C. glabrata* transporters are identified that mediate the uptake of lactate and/or acetate, it is unlikely that they play a crucial role in the tolerance to these acids (Vieira et al., 2010; Mota et al., 2015; Lourenco et al., 2018). *Candida* species can metabolize certain amounts of SCFA. This explains why the antifungal effect of several SCFA (acetate, butyrate, propionate, etc.) was shown to be concentration-dependent (Guinan et al., 2019). In *C. albicans*, SCFA can inhibit growth, germ tube formation, hyphae formation, hyphae attachment and reduce the metabolic activity of fungal cells in a biofilm. The effect of lactic acid on hyphae formation is controversial. Although it has been reported that certain lactobacilli can inhibit hyphae formation, the role of lactic acid in the process is not yet clear. Recent research showed that there is no clear correlation between the level of lactic acid produced and the level of inhibition. On the contrary, for D-lactic acid an inverse correlation was observed (Allonsius et al., 2019). Most of the effects of weak acids on microbial processes, are partially caused by inducing acidic external conditions due to the dissociative properties of SCFA in addition to currently unknown mechanisms (Guinan et al., 2019). Figure 3 illustrates the modes in which fatty acid metabolites can inhibit *Candida* pathogenicity. The inhibition of *C. albicans* growth is unlikely due to a change in environmental pH levels. *C. albicans* is capable of actively neutralizing acidic environments so SCFA-induced changes in environmental pH could not significantly affect their growth (Vylkova et al., 2011; Fan et al., 2015; Guinan et al., 2019). An important conclusion that can be drawn from various studies is that an acidic environment is needed for the antimicrobial activity of SCFA (Lai et al., 2009; Aldunate et al., 2015; Lourenco et al., 2018; Guinan et al., 2019). In neutral conditions they exist in anion form, but no longer exhibit inhibitory effects. Unlike many other weak acids, inhibition of *Candida* growth by lactic acid is controversial. In various studies, the inhibitory effect of lactic acid on growth appears to be minor (Moosa et al., 2004; Kasper et al., 2015; Lourenco et al., 2018), while in others, a clear effect is reported (Krasner et al., 1956; Kohler et al., 2012). In these studies, no distinction was made between both enantiomers of lactic acid. Since *Candida* species can mobilize lactate and acetate even when glucose is present, a possible explanation could be that lactate is rapidly metabolized while the metabolization of acetate may occur much more slowly giving it the time to exert its inhibitory effects (Childers et al., 2016; Lourenco et al., 2018).

As previously stated, the mechanisms by which SCFA exert their antifungal effects are not completely known yet. Various theories implicate intracellular acidification, accumulation of anions, ATP depletion and perturbation of the plasma membrane (Bracey et al., 1998; Guldfeldt and Arneborg, 1998; Stratford and Anslow, 1998; Mollapour et al., 2008; Ullah et al., 2012). To what extent each mechanism contributes to combined antifungal effects of various weak acids is not clarified yet. In *S. cerevisiae*, the lipophilicity of weak acids correlates with the acidification rate of the cytosol, which confirms that weak acids need to diffuse over the plasma membrane to be toxic, and that not the initial acidification but rather the ability of the fungal cell to restore the intracellular pH is an important determinant for growth inhibition (Purschke et al., 2012). The longer the chain length of a weak acid, the higher the lipophilicity and its toxicity. The mechanism to restore the cytosolic pH seems specific for each weak acid. For acetic acid, the activity of Pma1p, a plasma membrane H^+^-ATPase, is crucial for its resistance and long-term acidification is the major mechanism by which this weak acid inhibits growth. Although Pma1p activity is increased during acetic acid-stress, the capacity of the H^+^-ATPase as well as the ATP availability are probably not limiting. Acetic acid is as anion not very toxic. Its effect on the membrane integrity of fungi is not yet fully clear (Ullah et al., 2012). Acetic acid does not significantly affect membrane integrity in *S. cerevisiae* while Mira et al. (2010) reports that weak acids like acetic acid or lactic acid can cause membrane perturbation. This effect on membrane function and permeability may increase the uptake of azoles and explain the synergistic effect that is seen between acetic/lactic acid and azoles on the growth of *Candida* cells (Lourenco et al., 2018). *Candida* species have a better external pH adaptability than *S. cerevisiae*, and can therefore survive in environments between pH 2-10. This adaptability can potentially be explained by their broader cytosolic pH range. The cytosolic pH range of *S. cerevisiae* is between 6.0 and 7.0 while in *C. albicans* the intracellular pH range *in vivo* is wider, ranging between 5.8 and 9 (Cassone et al., 1983; Kaur et al., 1988; Stewart et al., 1988, 1989; Rabaste et al., 1995; Liu and Kohler, 2016; Tournu et al., 2017; Rane et al., 2019). Their growth is also less affected by an acidic cytosol compared to *S. cerevisiae*. Rane et al. (2019) it was shown that *C. albicans* cells with very acidic cytosols (pH ≤ 5.5) only show minimal growth defects. So SCFA probably exert their inhibitory effect on *Candida* species not by intracellular acidification alone. To better understand how exactly weak organic acids inhibit *Candida* cells, Cottier et al. (2015) looked at genomic and transcriptomic changes caused by lactic, acetic, propionic and butyric acid. They found that each organic acid triggers the expression of unique combinations of genes. Despite the differences in the induced responses, all the organic acids regulated the same sixteen genes at all-time points and independent of the pH. Thirteen of these genes (*CFL2*, *MP65*, *PIR1*, *ASR1*, *FET3*, *DAG7*, *GDH3*, *COI1*, *FRP1*, *6311*, *ICL1*, *FTR1*, *CAN1*) were up-regulated and three genes (*RPL13*, *HSP90*, *FTR2*) were down-regulated (Cottier et al., 2015). There are large similarities with the transcriptional response to reactive oxygen species and seven out of sixteen genes (*CFL2*, *COI1*, *FRP1*, *PIR1*, *FET3*, *FTR1*, *FTR2*) are involved in the regulation of iron homeostasis (Lan et al., 2004; Chen et al., 2011; Cottier et al., 2015). Cottier, Tan (Cottier et al., 2015) also found that all weak organic acids decreased the intracellular iron levels of *C. albicans* cells by approximately 60%. However, restoring normal intracellular iron levels by using a mutant that imports more iron did not significantly affect the inhibitory effect of butyric acid. So, the growth inhibition caused by lactic, acetic, propionic, and butyric acid cannot be explained by an intracellular iron drop alone. Still, iron is an essential micronutrient for *C. albicans* (Ramanan and Wang, 2000). It is critical for its growth, competition with other microbiota and interaction with the host (Ramanan and Wang, 2000; Purschke et al., 2012). Hence, the fact that weak organic acids decrease the availability of this important micronutrient calls for further research. Besides iron homeostasis, weak organic acids also had an effect on the expression of genes involved in host interaction, glycolysis, the biosynthesis of ATP, ergosterol, arginine and RNA and the biogenesis of ribosomes (Cottier et al., 2015). All acids down-regulated genes involved in RNA synthesis and ribosome biogenesis, especially during longer exposure. This caused a significant reduction in total RNA and the ratio ribosomal RNA/total RNA in *C. albicans* cells (Cottier et al., 2015). An overall down-regulation of transcription and translation is very typical for stress responses in general, and can be seen across a variety of microbial species (Lempiäinen and Shore, 2009). These data suggest that weak organic acids push *C. albicans* cells in a metabolic state similar to starved cells, in which the rates of transcription, translation and growth are low (Uppuluri and Chaffin, 2007). Which other mechanisms are contributing to the inhibiting effect of SCFA and to which extent, still needs to be elucidated.

MCFA are fatty acids with a chain length of 6-12 carbon atoms [caproic (C6), heptanoic (C7), caprylic (C8), non-anoic (C9), capric (C10), undecanoic (C11), and lauric (C12) acid]. In multiple studies, their antifungal effect on *Candida* species were demonstrated (Bergsson et al., 2001; Murzyn et al., 2010; Takahashi et al., 2012; Clitherow et al., 2020; Lee et al., 2020; Suchodolski et al., 2021). In Clitherow, Binaljadm (Clitherow et al., 2020), effects of all MCFA were tested on wild-type strains (SC5314, BWP17) and an azole-resistant strain (CAR17) of *C. albicans* and on other *Candida* species. Strain SC5314 and BWP17 were significantly inhibited in growth by heptanoic, caprylic and non-anoic acid. Caprylic and non-anoic acid even showed similar levels of inhibition to fluconazole and miconazole. Strain CAR17 was significantly inhibited in growth by MCFA ranging from C6-C10. Non-anoic acid also significantly inhibited the growth of *C. auris*, *C. tropicalis*, and *C. glabrata*. The effect of the MCFA were also tested on the biofilm viability of strain SC5314 (Clitherow et al., 2020). In contrast to the growth experiment, the three longest MCFA (capric, undecanoic and lauric acid) killed the most cells in the biofilm while heptanoic, caprylic and non-anoic acid were much less effective in reducing biofilm viability. The inhibition of MCFA on *C. albicans* growth and biofilm formation is confirmed by various other studies, however no consensus is reached on which MCFA are most potent (Bergsson et al., 2001; Murzyn et al., 2010; Takahashi et al., 2012; Clitherow et al., 2020; Lee et al., 2020).

*S. boulardii*, a commonly used probiotic yeast, produces caproic, caprylic, and capric acid. Murzyn et al. (2010) identified capric acid as the most effective antifungal MCFA that *S. boulardii* produced. It was the only MCFA of the three that inhibited the filamentation and adhesion of *C. albicans*. in *C. albicans, HWP1*, *INO1*, and *CSH1* expression were decreased upon capric acid treatment. *HWP1* encodes a hyphal wall protein that is important for both filamentation and adhesion (Nobile et al., 2006; Ene and Bennett, 2009). *INO1* encodes an enzyme required for the synthesis of GPI-anchored glycolipids, which is important during adhesion (Mille et al., 2004). *CSH1* encodes a protein that is important for the cell surface hydrophobicity of fungal cells, which increases *C. albicans* virulence by increasing adhesion to epithelial cells and are rendering *C. albicans* cells more resistant to phagocytes (Singleton et al., 2001, 2005). It is suspected that MCFA might exhibit their antifungal activity via mimicking the quorum-sensing molecule farnesol (Lee et al., 2020). Farnesol is a sesquiterpene produced by *C. albicans* that blocks filamentation at high cell densities in an autocrine manner (Ramage et al., 2002; Décanis et al., 2011). Various arguments state this claim. First, MCFA and farnesol are structural very similar. Secondly, heptanoic and non-anoic acid repress the same hypha- and biofilm-related genes (*HWP1*, *ALS3*, *ECE1*, and *UME6*) as farnesol (Lee et al., 2020). The genes that *S. boulardii*-produced capric acid downregulates (*HWP1*, *INO1*, and *CSH1*), are also downregulated by farnesol (Ramage et al., 2002; Cao et al., 2005; Murzyn et al., 2010). Lastly, after addition of MCFA to *C. albicans* cells, the production of farnesol is lower (Lee et al., 2020).

## Use of Fatty Acid Metabolites in Therapy

**FIGURE 4:**
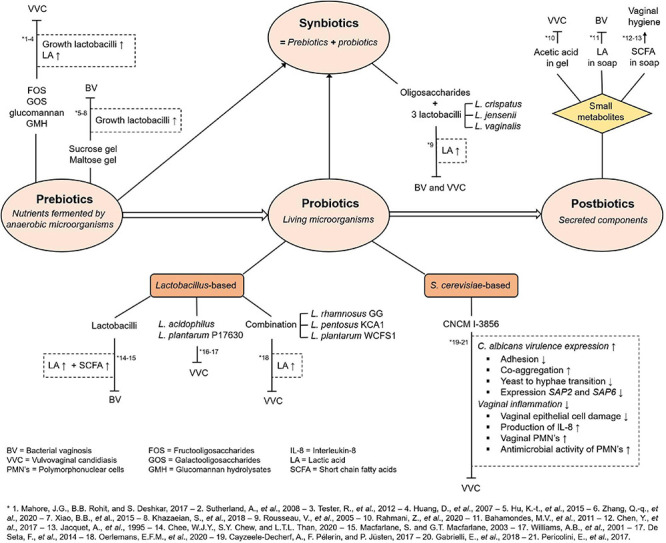
Overview of involvement of fatty acid metabolites in possible treatment of vaginal infections. Abbreviations and short references are given in the figure and designated by an asterisk. Details as well as full references are given in the main text.

Fatty acids can play a significant role in treatment of vaginal infections. Figure 4 schematizes the therapy strategies that rely on pre-, pro-, and postbiotics.

### Fatty Acid Producing Probiotics

#### *Lactobacillus*-Based Probiotics

As previously mentioned, *Lactobacillus* species are the most prevalent microbial species in the vaginal microbiome of most women and are the prime producers of vaginal metabolites, especially lactate (Redondo-Lopez et al., 1990). Because of this vaginal dominance and its promising antimicrobial effects as a probiotic in the gut, it is therefore no surprise that *Lactobacillus*-based probiotics are the focus for development of probiotic treatments for vaginal dysbiosis. In the context of vaginal disease, it has been shown that lactobacilli can decrease the infectivity of bacterial species associated with BV such as *Gardnerella vaginalis*, *Atopobium vaginae* and *Clostridium perfringens* by the production of lactate (Aldunate et al., 2015). However, the potential of lactobacilli to treat vaginal dysbiosis is strongly species dependent as the overall fatty acid production by *Lactobacillus* species depends on the individual potential of strain (Macfarlane and Macfarlane, 2003; Chee et al., 2020). Additional to the research on the potential of lactobacilli in the treatment of BV, many studies report the effectiveness of several *Lactobacillus* species against VVC (Williams et al., 2001; De Seta et al., 2014; Oerlemans et al., 2020). However, the role of lactobacilli as a probiotic to treat VVC is not univocal (Tachedjian et al., 2017). To date contradicting results keep arising on whether lactate produced by the lactobacilli has, at low pH, the ability to inhibit *Candida* growth and hyphal formation (Krasner et al., 1956; Lee et al., 1975; Morales and Hogan, 2010; Han et al., 2011). These contradicting results may be explained by the lactic acid tolerance of the tested *Candida* species (Cunha et al., 2017).

In the last two decades, various research teams investigated the potential of different *Lactobacillus* species as prophylactic therapy or treatment of VVC. Dating back to 2001, the research team of Williams et al. (2001) already investigated the probiotic effects of weekly intravaginal application of *Lactobacillus acidophilus* as a prophylactic treatment for VVC. It showed to have the same prophylactic capacities as the drug clotrimazole, one of the most commonly used imidazoles for VVC treatment (Sobel, 2014). Another study investigated the effect of intravaginally administered *Lactobacillus plantarum* P17630 on the reduction of VVC after conventional therapy with clotrimazole (De Seta et al., 2014). Women treated with *Lactobacillus plantarum* P17630 showed a significant increase in vaginal lactobacilli count. Moreover, the physiological pH was restored to stable levels and the women experienced significantly less discomfort, such as burning and itching after 3 months. However, once again no further investigation was executed to obtain insights into the role of lactate and SCFA in this treatment. More recently, a proof-of-concept study, performed by Oerlemans et al. (2020), formulated a gel-based system carrying three different *Lactobacillus* species, *L. rhamnosus* GG, *L. pentosus* KCA1, and *L. plantarum* WCFS1, and investigated its effectivity against VVC. During *in vitro* experiments, lactic acid production of several individual lactobacilli strains was evaluated and shown to display widespread concentration range from 2.78 g/L (*L. parabuchneri* AB17) to 20.22 g/L (*L. pentosus* KCA1). *L. rhamnosus* GG produced the highest amount of L-lactic acid (17.27 g/L) while *L. plantarum* WCFS1 produced the highest amount of D-lactic acid (7.68 g/L). Additionally, the latter strain inhibited *C. albicans* growth to the largest extent, implying that high amounts of D-lactic acid cause the greatest *C. albicans* growth inhibition. This result should, however, be interpreted with caution. As discussed on several occasions in the previous paragraphs, the ratio L- and D-lactic acid should be considered when selecting optimal probiotic strains. Although both enantiomers seem to have similar effects on the immune system and D-lactic acid was hypothesized to inhibit *Candida* growth further, it also negatively affects specific bacterial and viral pathogenesis to a lesser extent than L-lactic acid and is toxic to the host at high concentrations. The *in vivo* supplementation of the vaginal gel containing the three previously mentioned *Lactobacillus* strains, administered daily over a period of 10 days, proved to be effective against VVC in almost 10 out of the twenty participants.

To date, various *in vitro* studies and clinical trials using *Lactobacillus*-based probiotics as a treatment against VVC show promising results. However, the anti-*Candida* effect of certain probiotic lactobacilli strains are variable and highly species-specific. Therefore, it is impossible to extrapolate the probiotic tendencies and characteristics from one *Lactobacillus* species to another without extensive *in vitro* and *in vivo* studies.

#### *S. cerevisiae*-Based Probiotics

At this moment, most well-characterized and used microorganisms as probiotics are bacterial species of the genera *Lactobacillus* and *Bifidobacteria*. However, the last couple of years the interest in fungi-based probiotics is growing (Bermudez-Brito et al., 2012). This is not surprising since they offer important advantages over bacterial probiotics. Fungi have a unique cell wall construction. It consists of two layers of which the inner layer is composed of chitin, 1,3-β-glucan and 1,6-β-glucan and the outer layer contains mannan (Lipke and Ovalle, 1998). This structure allows fungi to easily survive passage through the gastrointestinal tract as many probiotics are taken orally (Banik et al., 2019). Moreover, fungal probiotics are resistant to antibiotics. This means that, in addition to the fact that their antibiotic resistance profile does not need to be investigated, they can also be used in patients taking antibiotics, which is an important risk factor for VVC (Xu et al., 2008; Gaziano et al., 2020). The most common fungal probiotic on the market is *Saccharomyces cerevisiae* var. *boulardii* (Sen and Mansell, 2020). This yeast is currently being used in the treatment of chronic and acute gastrointestinal diseases like inflammatory bowel disease, bacterial and rotaviral diarrhea (Madsen, 2001; Kelesidis and Pothoulakis, 2012). Only recently, it has been demonstrated that *S. cerevisiae*-based probiotics show potential for treatment of not only VVC, but also BV (Cayzeele-Decherf et al., 2017; Pericolini et al., 2017; Gabrielli et al., 2018; Sabbatini et al., 2018). *S. cerevisiae* strain CNCM I-3856 reduced the *C. albicans* vaginal load in women with VVC and increased the clearance of *C. albicans* from the vagina in mice (Cayzeele-Decherf et al., 2017; Pericolini et al., 2017). *In vitro*, this strain inhibited *C. albicans* adhesion to vaginal epithelial cells, induced *C. albicans* co-aggregation, inhibited *C. albicans* germ-tube and hyphae formation and reduced vaginal epithelial cell damage (Pericolini et al., 2017). Strain CNCM I-3856 was also shown to suppress the expression of secretory aspartyl proteinase (Sap) genes *SAP2* and *SAP6* in *C. albicans* both *in vitro* and in mice (Pericolini et al., 2017). Saps are important virulence factors of *C. albicans* as they play a major role during adhesion to and invasion of the host cells (Naglik et al., 2003). The suppression of Sap2 and Sap6 is specifically relevant for treatment of VVC due to their proinflammatory nature since vaginal inflammation is crucial in the pathogenesis of vaginal *Candida* infections (Pericolini et al., 2015; Vecchiarelli et al., 2015). Strain CNCM I-3856 also showed other therapeutic effects like reducing interleukin-8 production (IL-8), reducing the number of vaginal polymorphonuclear cells (PMNs) and enhancing the antimicrobial activity of PMNs (Gabrielli et al., 2018). IL-8 is a key cytokine during inflammatory processes. It recruits PMNs, which release proinflammatory substances by degranulation (Lacy, 2006; Gabrielli et al., 2018). These cells also have the capacity to produce diverse antimicrobial proteins and enzymes to kill small engulfed microorganisms, and release reactive oxygen species and cytokines to kill microorganisms extracellularly (Lacy, 2006). By reducing IL-8 and the number of PMNs in the vagina, *S. cerevisiae* can dampen local inflammation while maintaining or even enhancing the antimicrobial activity of the PMNs (Gabrielli et al., 2018). Current research on vaginal *S. cerevisiae*-based probiotics is not focused on the beneficial effects of SCFA. However, this could be very interesting since one of the mechanisms by which *S. cerevisiae* potentially exerts its probiotic effects in the gut, is the production of SCFA due to their immunomodulatory properties (Schneider et al., 2005; Ratajczak et al., 2019; Sen and Mansell, 2020). It is also demonstrated in several studies including our own unpublished research, that acetate, butyrate and propionate have an antifungal effect on *Candida* species (Nguyen et al., 2011; Yun and Lee, 2016; Lourenco et al., 2018). Focusing on high SCFA production together with other mechanisms-of-action during the development of vaginal *S. cerevisiae*-based probiotics could be advantageous.

### Prebiotics Stimulating Production of Fatty Acid Metabolites

Originally a prebiotic was defined as a non-digestible food ingredient that selectively stimulates the growth and/or activity of bacteria in the colon, and therefore improves the health of the host (Gibson and Roberfroid, 1995). Later, in 2008, this definition was refined by the International Scientific Association of Probiotics and Prebiotics (ISAPP) as a compound fulfilling three main criteria. Firstly, the compound should not be absorbed in the gastrointestinal tract and needs to be resistant to the acidic pH of the stomach and hydrolysis by mammalian enzymes. Secondly, the prebiotic needs to be fermented by intestinal microbiota. And thirdly, it needs to selectively stimulate the growth and/or the activity of the intestinal bacteria and improve the host health (Gibson et al., 2010). Various soluble fibers match these criteria and are fermented by anaerobic microbiota in the colon, to produce weak acids as byproducts (den Besten et al., 2013; McLoughlin et al., 2017). These fatty acids cause a drop in the colonic pH, and therefore promote the growth of some bacteria like *Lactobacillus* and *Bifidobacterium*. These bacteria are known as potent fatty acid producers that can produce other antimicrobial agents like hydrogen peroxide, bacteriocins and related substances for maintaining healthy immune responses (Basu et al., 2006; Simpson et al., 2006; Cocolin et al., 2007a). Fructooligosaccharides (FOS), galactooligosaccharides (GOS), isomaltooligosaccharides (IMO), xylooligosaccharides (XOS), lactulose, inulin, polydextrose and lactitol are categorized as prebiotics (Stowell, 2006). The type of prebiotic fiber results in production of different SCFA concentrations (den Besten et al., 2013). Short chain molecules with a low degree of polymerization, like oligosaccharides (e.g., FOS, GOS, inulin) are readily fermented and result in a higher SCFA yield compared to longer-chain polysaccharide soluble fibers, such as pectin (Slavin, 2013). Moreover, SCFA production is influenced by the composition of the microbiota in the colon, the site of substrate fermentation and by gut transit time (Lewis and Heaton, 1997; Wong et al., 2006).

When investigating the potential of using prebiotics against vaginal infections its definition requires re-formulation, as this was devised specifically for application in the gastro-intestinal tract. We can define prebiotics in the context of vaginal health, as ingredients that, when applied to the vaginal cavity, result in specific changes in the composition and/or activity of the genital microbiota, thereby conferring a specific benefit to the host. Most often these ingredients are nutrients that specifically allow growth of beneficial microbes which can thereby outcompete the pathogen (Rousseau et al., 2005). Since lactobacilli are the dominant microbiota in the vagina of most women, prebiotics can stimulate the growth of the body’s native lactobacilli, and are therefore capable of maintaining, restoring or optimizing the flora of the vaginal ecosystem (Al-Ghazzewi et al., 2007; Cocolin et al., 2007b; Ravel et al., 2011). In the first study with the use of prebiotics in vaginal environment, Rousseau et al. (2005) selected different prebiotic oligosaccharides, in combination with three different human vaginal *Lactobacillus* strains with probiotic properties, *L. crispatus*, *L. jensenii*, and *L. vaginalis*, to investigate the effect on pathogenic microorganisms like *C. albicans* and *Gardnerella vaginalis* which are often encountered in vaginal infections. Oligosaccharides FOS Actilight^®^ DP3, α-1,6/α-1,4 GOS and α-1,2/α-1,6/α-1,4 GOS (with α-1,6 and α-1,4 bonds similar to α-1,6/α-1,4 GOS) were consumed by the *Lactobacillus* strains resulting in a lactate concentration of 3–7,6 g/l after fermentation, while FOS Raftilose^®^ was fermented to a much lower extent and did not result in lactate production. The pathogenic microorganisms were unable to ferment these oligosaccharides FOS Actilight^®^ and α-1,6/α-1,4 GOS. Therefore, combining lactobacilli and oligosaccharides in a therapy can be a good method to prevent vaginal infections (Rousseau et al., 2005). Unfortunately, little information exists on how to prevent or treat vaginal infections with prebiotics in the form of a cream, douche, spray, pessary or tablet. A vaginal bio-adhesive delivery system based on pectinate-hyaluronic acid microparticles for prebiotics and probiotics encapsulation was designed for better controlled drug release in the vaginal tract (Pliszczak et al., 2011). An *in vitro* study showed that FOS and GOS stimulate the growth of lactobacilli, generating lactate and resulting in lower pH, causing suppressing of growth of harmful species like *E. coli* and potentially *C. albicans* in the vaginal ecosystem (Mahore et al., 2017). Other studies show that the prebiotics glucomannan and glucomannan hydrolysates (GMH) promote the growth, metabolism and antimicrobial properties *Lactobacillus* strains, with an increased inhibition of vaginal *Candida* species and link this to the produced lactic acid (Huang et al., 2007; Sutherland et al., 2008; Tester et al., 2012). Moreover, introduction of glucomannan hydrolyzates pessaries directly in the vagina showed an improvement of vaginal health (Al-Ghazzewi and Tester, 2016).

Animal trials indicate that the topical application of maltose gel and sucrose gel in the vagina of rhesus macaques can stimulate the growth of *Lactobacillus* species (Hu et al., 2015; Zhang Q.-Q. et al., 2020). Female rhesus macaques can be used as a good animal model to study vaginal microbiota-associated diseases, since their vagina is colonized by anaerobic bacteria which are normally associated with BV in women (Spear et al., 2010; Chen et al., 2018). Two clinical trials showed a high cure rate of clinical symptoms associated with bacterial vaginosis when treating with sucrose gel (Xiao et al., 2015; Khazaeian et al., 2018). A disadvantage when using sucrose gel is its instability at low pH. As far as we know, the combination of the vaginal probiotic *S. cerevisiae* and prebiotics have not been investigated.

### Fatty Acid Metabolites as Postbiotics

Apart from the well-known probiotics and prebiotics, also alternative approaches for using microbes to confer a health benefit to the host, are being investigated. Postbiotics are defined as soluble products secreted or released by and resulting from microbial metabolism that have a beneficial effect on the host (Teame et al., 2020). There is ongoing discussion on which type of molecules can be classified under this denominator. In some cases, postbiotics are seen as any product released by microbes that confers a benefit to the host, including proteins, vitamins, SCFA, polysaccharides and even cell wall fragments and lysates (Zolkiewicz et al., 2020). According to other researchers, these products of microbial origin must be further subdivided into two categories. The structural components of the cells, such as inactivated cells or cell fragments, are termed parabiotics, while secreted components are classified as postbiotics (Teame et al., 2020). In any case, it seems to be true that not only living organisms can stimulate host health, also metabolites can do so and are thus exploited commercially. Many types of secreted molecules can be considered as potential postbiotics, such as peptides, proteins and other small metabolites. An example of microbial peptides that confer benefits to the human host are bacteriocins, which are small antimicrobial peptides that are synthesized by various *Lactobacillus* species and can inhibit local pathogen overgrowth (Perez et al., 2014). More interesting in the context of this review, are the small molecules considered as postbiotics, such as neurotransmitters and SCFA. Weak organic acids have been used as supplements in wash liquids and intimate soaps (Jacquet et al., 1995; Chen Y. et al., 2017). Presence of lactic acid was shown to mitigate recurrence of BV after treatment with metronidazole (Bahamondes et al., 2011). Acetic acid formulations were shown to relieve VVC to some extent (Rahmani et al., 2020). Apart from these individual manuscripts, not much has been reported on the use of postbiotics to treat vaginal infections. However, the main benefit of postbiotics compared to probiotics is that no living micro-organisms need to be ingested or applied. This way, no harm can be inflicted by probiotic overgrowth and infection, as is reported in some exceptional cases involving critically ill patients (Kara et al., 2018; Castro-Gonzalez et al., 2019; Fadhel et al., 2019). Furthermore, postbiotics are more predictable and more easily standardized. Also transport and preservation is less critical compared to probiotics (Zolkiewicz et al., 2020).

## Conclusion

It seems evident from the information collected in this review article that fatty acid metabolites play an important role in vaginal health. These metabolites depict a close relationship to the local microbiome, as both factors can influence each other’s composition directly and indirectly. It thus makes sense that further elucidating the metabolome and its role in specific dysbiotic states can lead to insights in the cause, expression and possible treatment of vaginal infections. Research devoted specifically to the vaginal niche will allow the identification of components of the vaginal micro- or metabolome that link to onset of infections. Altering the microbial or metabolic composition of the vagina can therefore be a promising therapy strategy to prevent or cure infections such as BV or VVC.

## Author Contributions

SB, MS, LD, IP, and PV wrote the manuscript. All authors aided in proofreading and improving the manuscript draft.

## Conflict of Interest

The authors declare that the research was conducted in the absence of any commercial or financial relationships that could be construed as a potential conflict of interest.

## Footnotes

1 http://blast.ncbi.nlm.nih.gov/Blast.cgi

2 http://www.cbs.knaw.nl/collections/BioloMICSSequences.aspx
