# Supplementary material for: The Role of Fatty Acid Metabolites in Vaginal Health and Disease: Application to Candidiasis
Source: Front Microbiol. 2021 Jul 2;12:705779. doi: 10.3389/fmicb.2021.705779 (PMC8282898; doi:10.3389/fmicb.2021.705779)
Supplement: Supplementary file 1 [file Table_1.DOCX]

| Vaginal microbes with unreported fatty acid metabolite production |
| --- |
| *Aerococcus* |
| *Alternaria* |
| *Aspergillus* |
| *Cladosporium* |
| *Citrobacter* |
| *Davidiellaceae* |
| *Eggertthella* |
| *Eurotium* |
| *Faclamia* |
| *Finegoldia* |
| *Malassezia* |
| *Microvirga* |
| *Parvimona* |
| *Peptinophilis* |
| *Providencia* |
| *Rhizobiales* |
| *Rhodaturola* |
| *Salmonella* |
| *Sneathia* |
| *S. cerevisiae* |

**Supplemental table 1. List of microbes without reported vaginal production of fatty acid metabolites**
